# Characteristics Associated with Utilization of Asthma-Related Traditional Chinese Medicine Services among Asthma Children in Taiwan: A Nationwide Cohort Study

**DOI:** 10.1155/2015/108961

**Published:** 2015-04-20

**Authors:** Shiou-Ian Lin, Tung-Hu Tsai, Yiing-Jenq Chou, Nicole Huang

**Affiliations:** ^1^Institute of Traditional Medicine, National Yang-Ming University, No. 155, Section 2, Li-Nong Street, Taipei 112, Taiwan; ^2^Graduate Institute of Acupuncture Science, China Medical University, No. 91 Hsueh-Shih Road, Taichung 404, Taiwan; ^3^Department of Education and Research, Taipei City Hospital, No. 145 Zhengzhou Road, Datong District, Taipei 103, Taiwan; ^4^Institute of Public Health, School of Medicine, National Yang-Ming University, No. 155, Section 2, Li-Nong Street, Taipei 112, Taiwan; ^5^Institute of Hospital and Health Care Administration, School of Medicine, National Yang-Ming University, The Medical Building II, No. 155, Section 2, Li-Nong Street, Taipei 112, Taiwan

## Abstract

*Introduction*. Previous studies have demonstrated the advantages of TCM use among asthmatic children. However, there is a paucity of epidemiologic reports on features of TCM users among asthmatic children. This cohort study aimed to investigate child's, parent's, and provider's characteristics associated with the use of asthma-related TCM services among newly diagnosed asthmatic children. *Materials and Methods*. A nationally representative cohort of one million National Health Insurance beneficiaries was used. The newly diagnosed asthma children who received asthma medication from western medicine providers from 2005 to 2010 were selected as our sample for analysis. Generalized estimating equation was applied to identify the child's, parents', and provider's characteristics associated with the use of asthma-related TCM among the newly diagnosed asthmatic children. *Results*. Of 20,080 children who were enrolled and followed up for one year, 4,034 children used TCM for asthma-related treatment. Children with prior experience of TCM, pre-school and school aged children, boys, those with more severe asthma or poorer health, with higher income parents were more likely to use asthma-related TCM. Herbal medicine was the most common modality among asthmatic children. *Conclusions*. There were only 20% newly diagnosed asthmatic children using TCM. The findings may shed light on possible integration of TCM with western medicine services.

## 1. Introduction

Asthma is one most prevalent chronic condition among children. Global prevalence ranges from 1% to 18% across countries [1]. In addition to the large financial burdens, asthma also leads to serious health consequences and compromises life quality of children [1]. Poorly controlled childhood asthma may increase use of steroids, risk of emergency room visits or hospitalizations, persistently decrease lung function, or even lead to COPD later in adulthood [1]. According to the guideline published by the Global Initiative for Asthma (GINA), current western medications of asthma such as steroid, beta-2 adrenergic agonist, leukotriene modifier, theophylline, and anti-IgE therapies are the mainstream treatments of asthma. Recent empirical evidences also show good comparative effectiveness of integrating traditional Chinese herbal medicines to asthma management as complementary therapies, particularly among children [2–6]. Traditional Chinese herbal medicines such as ASHMI [2], mMMDT [3], Ding-Chuan [4], and STA-1 [5, 6] are proven safe and having a positive effect on symptoms and/or lung function in children.

Recently, the rising popularity of complementary and alternative medicine (CAM) has facilitated the research and development of scientifically sound CAM therapies. Numerous studies have investigated the utilization patterns of CAM among children [7–11] and found that CAM utilization is associated with children and parental characteristics, including age, gender, ethnicity, health conditions, household income, and parental use of CAM. Various CAM modalities children received included traditional Chinese medicine (TCM), herbal treatments, chiropractors, messages, and mind-body therapies [9]. However, not all CAM have been scientifically proven effective. Therefore, it is essential to investigate whether and to what extent some scientifically proven CAM therapies are used by children in addition to their mainstream medical therapies.

Of the existing literature, several studies focus specifically on CAM utilization of asthmatic children. The prevalence of CAM visits among asthmatic children ranged from 13% in Canada [12] to 89% in USA [13]. CAM treatments of asthma encompass many therapies, such as mind-body techniques, nutritional and herbal supplements, TCM, exercise, messages, and homeopathy [14]. CAM use in asthmatic children has been associated with children's age, asthma severity, parents' education level, parental income level, and insurance coverage [12, 13, 15–18].

Nevertheless, not only did characteristics of children and parents matter, but also the characteristics of their western medicine providers may influence their use of CAM [19–21]. Western medicine (WM) physicians may play a significant role in influencing parent's decision in seeking CAM therapies. WM providers' knowledge of, attitude towards, and practice of CAM may vary widely by their demographics, professional characteristics, and characteristics of the medical care system. Environmental factors such as availability of WM and CAM providers in the areas may create either a friendlier or a more hostile atmosphere for integration [22]. Hence, the role of providers in use of CAM among asthmatic children shall not be overlooked.

Furthermore, the existing findings on asthmatic children are mostly conducted in health care systems where CAM services are not covered by insurance, or in hospital settings. None of these studies are conducted in a health care system where a major domain of CAM, TCM, is covered by a social health insurance. It may be interesting to investigate the utilization pattern of asthma-related TCM among asthmatic children in the National Health Insurance (NHI) program in Taiwan, where TCM is comprehensively covered by the NHI program. This study aimed to investigate the use of TCM among the newly diagnosed asthmatic children and its associated characteristics of children, parents, and WM providers.

## 2. Materials and Methods

### 2.1. Data Source

The data source is the Taiwan National Health Insurance Research Database (NHIRD). The National Health Insurance program is a single-payer mandatory universal insurance system. In 2011, the enrollment rate was 99% of the total population [23]. It is well known for its comprehensive benefit coverage. It not only covers ambulatory and inpatient services of western medicine, but also reimburses TCM ambulatory care services. The NHI TCM coverage includes Chinese herbal medicine, acupuncture, traumatology, and massage. The dataset consists of individual's enrolment and claims information, including patients' identification number, gender, birthdate, date of service, diagnosis, medication, treatment procedure, and expenditures. The dataset also records provider's information, including gender, age, specialty of physicians, and ownership and accreditation level of physician's practice location. Individual and provider identifiers were encrypted before releasing to the researchers.

### 2.2. Study Design and Sample

This study was a retrospective cohort study. Of all the NHI enrollees in 2005 throughout Taiwan from the NHIRD, a representative cohort of 1 million randomly sampled enrollees was used. The newly diagnosed asthmatic children who were aged 0–18 years and received his/her primary diagnosis of asthma and asthma medication from western medicine providers from 2005 to 2010 were included for analyses. Asthma medications in western medicine were defined according to the GINA guideline. The children who had received any diagnosis of asthma within the five years before the index year were excluded. International Classification of Diseases, Ninth Revision, Clinical Modification codes 493.xx were used to identify asthma children. A total of 20,080 children were newly diagnosed with asthma in 2005–2010. Each child had been followed for one year since his/her first diagnosis of asthma for any asthma-related TCM visit.

### 2.3. Variables

Asthma-related TCM visits were defined as visits of primary TCM diagnostic code of respiratory diseases (ICD-code: 460~466, 470~478, 480~488, 490~496, 500~508, 510~519). Asthma is a chronic respiratory disease and may coexist with or have other related respiratory conditions, such as respiratory tract infection [24], allergic rhinitis [25], chronic rhinitis [26], chronic bronchitis, and emphysema [27]. According to the fundamentals of TCM theories, literatures, and clinical practices, TCM normally treat diseases from a more holistic perspective. TCM users were defined as those who had any asthma-related TCM visits during the 1-year follow-up period. TCM nonusers refer to those without any asthma-related TCM visits during the 1-year follow-up period. Sensitivity analyses were also conducted for various definitions of asthma-related TCM visits. The results remained robust. Furthermore, specific types of asthma-related TCM modalities children received and the top 10 commonly prescribed Chinese herbal medicinal formulas during the follow-up period among the asthmatic children were also analyzed.

Characteristics of children, parents, and WM providers were compared between TCM users and nonusers. Child's age, gender, health status (any hospitalization prior to the index year), previous TCM experience, level of medical resources of residential location, asthmatic WM medication used, frequency of WM visits, and incident year of asthma were constructed. Asthma medications used included controller medications, oral and systemic corticosteroids. Controller medications include LABAs, xanthines, ICS, leukotriene modifiers, immunomodulators, and cromolyn sodium. Controller medications were used for controlling persistent asthma. Oral or systemic corticosteroids were used for quickly reliving asthma exacerbations. Level of medical resources was defined as densities of western medical doctors and TCM doctors. Density of TCM and WM doctors were defined as number of TCM/WM doctors per 10,000 population and classified into four categories: (1) high density of TCM doctors and high density of WM doctors, (2) high density of TCM and low density of WM doctors, (3) low density of TCM and high density of WM doctors, and (4) low density of TCM and low density of WM.

In addition, socioeconomic status of parents was defined using the insurance wage and category of the insured. For people with well-defined monthly wages, we categorized them into 3 groups: ≥40000 NTD, 20000–39999 NTD, <20000 NTD. For those without a clearly defined monthly wage, including fisherman and farmers, we categorize them in another group.

WM provider was defined as the most frequently visited western medicine doctor for asthma during the follow-up year. If the most frequently visited WM doctors were more than one, the provider was defined as the earliest one a child had visited. Characteristics of WM providers included WM physician's age, gender, specialty, ownership, and accreditation level of his/her practice location.

### 2.4. Statistical Analysis

Descriptive statistics were presented. A chi-square test was used to examine differences in characteristics between TCM and non-TCM users. To adjust for correlation among the care seeking behavior of children grouped into provider clusters, generalized estimating equation (GEE) was also applied to analyze the influence of children's, parental, and provider's characteristics on the concurrent use of TCM among the newly diagnosed children receiving WM medication. A two-tailed *P* value of less than 0.05 was considered statistically significant. SAS 9.3 was used for data management and statistical analysis.

## 3. Results

Of the 20,080 children who were enrolled and followed up for one year, 4,034 children (20.1%) had at least one asthma-related TCM visit; 16,046 (79.9%) did not.  Table 1 shows the distribution of TCM users and TCM nonusers. TCM use among the group of infants and toddlers was the lowest (12.0%), whereas TCM use among the group of previous TCM users was the highest (46.8%). The variations of TCM use among children treated by WM providers were small, ranging from 16.9% to 21.6%.

Table 2 shows that children's age, gender, health status, and visits of WM doctor were significantly associated with TCM use. Preschool and elementary school children were 1.72 times and 1.5 times more likely to use asthma-related TCM than infant and toddler. Boys had 12.0% higher probability of using asthma-related TCM than girls. Prior TCM experience was the strongest predictor of using TCM when children were diagnosed with asthma (OR: 6.33). Children who had been hospitalized in the previous year (OR: 1.16) or who had more frequent WM outpatient visits (OR: 1.15) were significantly more likely to have asthma-related TCM visits. Children who received oral or systemic corticosteroids were associated with a higher likelihood of TCM visits (OR: 1.08) at the borderline significance (95% CI: 1.00, 1.18). Children of parents with monthly insurable wage below 20,000 NTD or without regular monthly wage were 13.0–16.0% less likely to seek TCM care for asthma.

Factors such as physician's gender, age, specialty, ownership and accreditation level of facility, and density of WM and TCM doctors were not significantly associated with asthma-related TCM use. However, asthmatic children whose main WM physicians aged 41~50 years were associated with a significantly higher likelihood of using TCM (OR: 1.09) at the borderline significance level (95% CI: 1.00, 1.18). Children who were treated by pediatricians were also significantly more likely to use TCM (OR: 1.17) at the borderline significance level (95% CI: 1.00, 1.38). No statistically significant difference was observed among children living in locations with varying density levels of WM and TCM doctors.

Herbal medicine was the most commonly used TCM modulation (99.6%) among the newly diagnosed asthma patients.  Table 3 shows the top 10 commonly prescribed Chinese herbal formulas children used. Shin-Yi-Qing-Fei-Tang was the most commonly used herbal formula (21.4%). Shin-Yi-Qing-Fei-Tang composes* Magnolia liliiflora*,* Lilium brownie*,* Anemarrhena asphodeloides*,* Scutellaria baicalensis*, calcium sulphate,* Eriobotrya japonica*,* Cimicifuga foetida*,* Ophiopogon japonicus*,* Gardenia jasminoides*, and* Glycyrrhiza uralensis*. Xiao-Qing-Long-Tang was the second commonly used herbal formula (16.3%). Xiao-Qing-Long-Tang is composed of* Magnolia liliiflora*,* Lilium brownie*,* Anemarrhena asphodeloides*,* Scutellaria baicalensis*, calcium sulphate,* Eriobotrya japonica*,* Cimicifuga foetida*,* Ophiopogon japonicus*,* Gardenia jasminoides*, and* Glycyrrhiza Uralensis*. Cang-Er-Zi-San was the third commonly used herbal formula (14.5%). Cang-Er-Zi-San is composed of* Xanthium sibiricum*,* Magnolia liliiflora*,* Angelica dahurica*, and* Mentha haplocalyx*. Among three formulations, Xiao-Qing-Long-Tang had been scientifically proven for its effect on decreasing asthmatic Th2-related immune response and having the bronchodilating effect [28–30]. A formula of modified Cang-Er-Zi-San: Shi-Bi-Lin had been studied for its effect on relieving symptoms of nose blockage [31]. The major component of Shin-Yi-Qing-Fei-Tang, Magnoliae flos (Shen et al.), had been studied of its treatment effect on sinusitis and asthma [32].

## 4. Discussions

This study has revealed several statistically significant patterns regarding TCM utilization among asthmatic children under the NHI program in Taiwan. First, despite the minimal financial barriers to TCM services under the NHI program in Taiwan, only 20.1% of the newly diagnosed asthmatic children used asthma-related TCM, which was among the lower bound of estimates observed in other societies. This low prevalence of TCM use reflects a large room for improvement in integrating TCM modalities with the mainstream asthmatic care. Second, substantial variations were observed across different children groups. Consistent with previous literatures, in addition to preschool age [12] and boys, children with prior experience with TCM, more severe asthma, or poorer health [12, 17, 18, 33] were more likely to seek TCM care for better control of their asthma conditions. Unexpectedly, under the NHI program, socioeconomic differences were also observed in the use of TCM care among asthma children. Children of higher income parents were more likely to seek TCM care for asthma [7]. Third, the marginal statistically significant odds ratios observed across all provider characteristics ring a warning bell. The findings suggest that the utilization pattern of TCM among newly diagnosed asthmatic children did not vary dramatically across provider characteristics. More importantly, the findings imply that the integration of TCM care remained universally low among all WM providers. More specifically, previous use of TCM was the strongest influencing factor of TCM use. Similarly in Norway, adolescents' previous visits of CAM were also the strongest predictor of future CAM visits in the Young-HUNT studies [34]. Asthmatic patients accustomed to TCM therapies or believing in TCM treatments may lead to further integrative use of WM and TCM. Hence, it is important to increase awareness of or positive attitudes towards scientifically proved TCM care and build trust of TCM among asthmatic children and parents. More effective promotion or educational strategies or program about TCM may help to reduce nonfinancial barriers to TCM services.

Furthermore, in spite of low financial barriers to TCM care under the NHI program, children with higher income parents were significantly more likely to receive TCM care in addition to their WM asthmatic treatments. This may also be due to other barriers. A possible barrier is that parents' workload may be so hard that they did not have spare time to bring their children to visit other doctors. Further studies of other nonfinancial barriers (time or travel costs) are needed to facilitate better or more efficient integration of TCM and WM asthmatic treatments.

All of the provider factors studied are not significantly associated with TCM use among asthmatic children. Most importantly, the persistently low utilization of TCM across WM providers of different characteristics may reflect that the issue of integration of TCM and WM is generally overlooked in routine management of pediatric asthma. Furthermore, limited availability of clinical trials may be one plausible barrier to better integration of TCM and WM. Whether or not to use TCM to treat or control asthma may heavily depend on therapeutic effects and safety of TCM. Therefore, more clinical trials should be encouraged in this regard.

This study has several strengths. First, using the National Health Insurance data of a large national representative sample in Taiwan can help us avoid some of the known shortcomings of survey data. The National Health Insurance covered both TCM and WM universally. Using the medical records from National Health Insurance data may ameliorate the selection bias from the survey data. Second, in contrast to other studies on this topic, the large sample size and relatively comprehensive data allow us to go beyond prior research by investigating not only children and parental characteristics, but also WM provider characteristics in their influences on the utilization of TCM care among the newly diagnosed asthma children. Third, taking advantages of the detailed information on TCM modalities and herbal medications in the NHI databases, we were able to identify the common TCM medications and therapies prescribed to the newly diagnosed asthmatic children in Taiwan. The findings may serve as important references in clinical management of pediatric asthma and for future effectiveness researches in TCM.

Several limitations should also be noted. First, the NHI database only allows us to estimate the use of TCM services covered by the NHI program. The NHI program covers TCM services provided by the NHI contracted TCM providers, including extracted TCM powder preparations, acupuncture, moxibustion, and traumatology manipulative therapy. However, other TCM services or products including crude herbal medicine, herb tea bags, pills, capsules, and other CAM modalities are not covered by the NHI program and these insurance noncovered services are self-paid by patients. The prevalence of people who had used any self-paid TCM or CAM service in one year was 6.05% [35]. Those self-paid TCM services or CAM services are not included for analyses in this study. Not being able to include self-paid TCM or CAM services in this study may lead to an underestimation of the CAM use among asthmatic children in Taiwan. However, as the CAM ranges widely, not many modalities have been proven effective scientifically. As the TCM services covered by the NHI program are well accepted CAM modalities and patient's costs in using these services are very low, studying the TCM utilization patterns under the NHI program may help to go beyond the previous investigations of general CAM uses. Second, a possible misclassification bias may be of concern. Because a claim may have up to 3 diagnoses, defining asthma-related TCM visits based on one set of respiratory diagnoses may be questionable. Therefore, sensitivity analyses were conducted on various definitions of TCM visits containing only diagnoses of respiratory diseases, any diagnoses of respiratory diseases, diagnoses of asthma, and diagnoses of asthma and bronchitis. All major statistical results from sensitivity analyses remained significant. Third, due to data limitations, using a single measure (i.e., the NHI program's payroll and occupation-based categories) to analyze the association between a parent's SES and the decision on TCM care did not allow us to fully explore this association. In addition, possible confounding biases such as knowledge level may also exist.

## 5. Conclusion

Adopting TCM care for asthma management among the newly diagnosed children in Taiwan was low. The low prevalence of TCM use is commonly seen across children treated by different characteristics of WM providers. This may suggest there is still room to promote collaboration between WM and TCM providers. Instead of targeting specific physician groups, putting more efforts generally in integration between TCM and WM in the management of pediatric asthma is needed. More efforts in improving understanding of TCM such as the educational programs of TCM and more research efforts in showing evidence-based effectiveness of TCM modalities may help to build more confidence or positive attitudes towards TCM among physicians and parents and to facilitate the integration TCM into the mainstream medicine in pediatric asthma management.

## Figures and Tables

**Table 1:** Distribution of TCM users and nonusers in asthmatic children by different characteristics of children, parents, and providers.

Child and parental characteristics	TCM user (%)	TCM nonuser (%)	Chi-square *P* value
Age			
Infant and toddler	286 (11.9)	2,114 (88.1)	<0.001^∗^
Preschool	1,779 (22.2)	6,223 (77.8)	
School	1,600 (21.5)	5,831 (78.5)	
Adolescent	369 (16.4)	1,878 (83.6)	
Gender			
Girls	1,675 (19.3)	6,990 (80.7)	0.019^∗^
Boys	2,359 (20.7)	9,056 (79.3)	
Previous use of TCM			
Yes	1,952 (12.5)	13,683 (87.5)	<0.001^∗^
No	2,082 (46.8)	2,363 (53.2)	
Previous hospitalization			
Yes	3,331 (19.7)	13,598 (80.3)	0.001^∗^
No	703 (22.3)	2,448 (77.7)	
Controller medication used			
Yes	2,662 (21.3)	9,836 (78.7)	<0.001^∗^
No	1,372 (18.1)	6,210 (81.9)	
Oral/systematic corticosteroid used			
Yes	1,851 (20.9)	7,000 (79.1)	0.010^∗^
No	2,183 (19.4)	9,046 (80.6)	
Number of outpatient visits in one year			
<3	2,161 (18.6)	9,431 (81.4)	<0.001^∗^
≥3	1,873 (22.1)	6,615 (77.9)	
Density of TCM and WM			
Low TCM and low WM	563 (19.6)	2,315 (80.4)	0.006^∗^
Low TCM and high WM	326 (19.0)	1,393 (81.0)	
High TCM and low WM	857 (18.7)	3,717 (81.3)	
High TCM and high WM	2,288 (21.0)	8,621 (79.0)	
Incident year			
2005	841 (18.7)	3,648 (81.3)	<0.001^∗^
2006	710 (18.7)	3,080 (81.3)	
2007	806 (19.8)	3,269 (80.2)	
2008	677 (21.7)	2,438 (78.3)	
2009	616 (23.2)	2,041 (76.8)	
2010	384 (19.7)	1,570 (80.4)	
Parent's insurable wage/category			
<20000 NTD	1,099 (18.6)	4,802 (81.4)	0.002^∗^
20000~39999 NTD	1,418 (20.5)	5,505 (79.5)	
≥40000 NTD	987 (21.6)	3,586 (78.4)	
Special group	530 (19.8)	2,153 (80.3)	
Provider characteristics			
Age			
≤40	1,772 (19.5)	7,336 (80.5)	0.108
41~50	1,672 (20.7)	6,391 (79.3)	
≥51	590 (20.3)	2,319 (79.7)	
Sex			
Female	691 (21.6)	2,515 (78.5)	0.024^∗^
Male	3,343 (19.8)	13,531 (80.2)	
Specialty			
Family medicine	271 (16.9)	1,329 (83.1)	0.008^∗^
Internal medicine	247 (19.8)	1,001 (80.2)	
Pediatrics	2798 (20.6)	10,815 (79.5)	
Other	718 (19.8)	2,901 (80.2)	
Ownership			
Private	3,694 (20.1)	14,729 (80.0)	0.649
Public	340 (20.5)	1,317 (79.5)	
Accreditation level			
Hospital	1,921 (20.8)	7,301 (79.2)	0.016^∗^
Clinic	2,113 (19.5)	8,745 (80.5)	

^∗^Statistically significance (*P*-value < 0.05).

**Table 2:** Children, parent, and provider characteristics associated with TCM uses.

Child's characteristics	Crude OR (95% CI)	Adjusted OR (95% CI)
Age (ref.: infant and toddler)		
Preschool	2.11 (1.85, 2.42)^∗^	1.72 (1.49, 1.99)^∗^
School	2.03 (1.77, 2.32)^∗^	1.50 (1.28, 1.75)^∗^
Adolescent	1.45 (1.23, 1.72)^∗^	0.91 (0.75, 1.11)
Sex (ref.: girls)		
Boys	1.09 (1.01, 1.17)^∗^	1.12 (1.04, 1.20)^∗^
Previous use of TCM (ref.: no)		
Yes	6.18 (5.73, 6.66)^∗^	6.33 (5.83, 6.87)^∗^
Previous hospitalization (ref.: no)		
Yes	1.17 (1.07, 1.29)^∗^	1.16 (1.05, 1.29)^∗^
Controller medication used (ref.: no)		
Yes	1.23 (1.14, 1.32)^∗^	1.07 (0.98, 1.16)
Systematic or oral corticosteroid used (ref.: no)		
Yes	1.10 (1.02, 1.17)^∗^	1.08 (1.00, 1.18)
Number of outpatient visits in one year (ref.: <3)		
≥3	1.24 (1.15, 1.33)^∗^	1.15 (1.06, 1.25)^∗^
Density of TCM and WM doctors (ref.: low TCM and low WM)		
Low TCM and high WM	0.96 (0.83, 1.12)	0.92 (0.77, 1.09)
High TCM and low WM	0.95 (0.84, 1.07)	0.90 (0.78, 1.03)
High TCM and high WM	1.09 (0.98, 1.21)	1.00 (0.89, 1.12)
Incident year (ref.: 2005)		
2006	1.00 (0.90, 1.12)	1.00 (0.89, 1.12)
2007	1.07 (0.96, 1.19)	1.03 (0.92, 1.15)
2008	1.21 (1.08, 1.35)^∗^	1.03 (0.91, 1.17)
2009	1.31 (1.16, 1.47)^∗^	1.17 (1.03, 1.33)^∗^
2010	1.06 (0.93, 1.21)	0.91 (0.79, 1.06)
Parental characteristics		
Parent's insurance wage (ref.: ≥40000 NTD)		
<20000 NTD	0.83 (0.76, 0.92)^∗^	0.84 (0.76, 0.94)^∗^
20000~39999 NTD	0.94 (0.85, 1.03)	0.93 (0.84, 1.02)
Special group	0.89 (0.80, 1.01)	0.87 (0.76, 0.99)^∗^
Provider's characteristics		
Age (≤40)		
41~50	1.08 (1.01, 1.17)^∗^	1.09 (1.00, 1.18)
≥51	1.05 (0.95, 1.17)	1.10 (0.97, 1.24)
Sex (ref.: female)		
Male	0.90 (0.82, 0.99)^∗^	0.90 (0.80, 1.02)
Specialty (ref.: family medicine)		
Internal medicine	1.21 (1.00, 1.47)	1.23 (0.99, 1.52)
Pediatrics	1.27 (1.11, 1.46)^∗^	1.17 (1.00, 1.38)
Other	1.21 (1.04, 1.42)^∗^	1.19 (1.00, 1.42)
Ownership (ref.: public)		
Private	0.97 (0.86, 1.10)	1.06 (0.92, 1.23)
Accreditation level (ref.: hospital)		
Clinic	0.92 (0.86, 0.98)^∗^	0.93 (0.85, 1.01)

^∗^Statistically significance (*P*-value < 0.05).

**Table 3:** Top 10 Chinese herbal medicines used.

Top 10	Herbal formulas	Frequency (%)
1	Shin-Yi-Qing-Fei-Tang	21.4
2	Xiao-Qing-Long-Tang	16.3
3	Cang-Er-Zi-San	14.5
4	Shin-Yi-San	13.9
5	Ma-Xing-Gan-Shi-Tang	12.4
6	Xing-Su-San	8.7
7	Ge-Gen-Tang	7.9
8	Yin-Qiao-San	7.4
9	Ding-Chuan-Tang	5.9
10	Zhi-Sou-San	5.5
